# Altered Expression of Three EGFR Posttranslational Regulators MDGI, MIG6, and EIG121 in Invasive Breast Carcinomas

**DOI:** 10.1155/2020/9268236

**Published:** 2020-04-20

**Authors:** Didier Meseure, Kinan Drak Alsibai, Sophie Vacher, Rana Hatem, Andre Nicolas, Celine Callens, Florence Lerebours, Ivan Bieche

**Affiliations:** ^1^Platform of Experimental Pathology, Institut Curie, F-75248 Paris, France; ^2^Department of Diagnostic and Theranostic Medicine, Institut Curie, F-75248 Paris, France; ^3^Unit of Pharmacogenomics, Department of Genetics, Institut Curie, F-75248 Paris, France; ^4^Department of Pathology, Cayenne Hospital Center, F-97306 Cayenne, France; ^5^Center of Biological Resources (CRB Amazonie), Cayenne Hospital Center, F-97306 Cayenne, France; ^6^Department of Medical Oncology, Institut Curie, Rene Huguenin Hospital, F-92210 Saint-Cloud, France; ^7^Faculty of Pharmaceutical and Biological Sciences, Sorbonne Paris Cité, Paris Descartes University, F-75006 Paris, France

## Abstract

Epidermal growth factor receptor (EGFR) signalling is a highly regulated process with a tight balance between receptor activation and inactivation in invasive breast carcinomas (IBCs) particularly in triple-negative carcinomas (TNC). Clinical trials using anti-EGFR therapies are actually performed although no activating alterations (mutations, amplifications, or rearrangements) of *EGFR* have been clearly recognized in order to identify new targeted modalities for IBCs. We explored mammary-derived growth inhibitor (MDGI), estrogen-induced gene-121 (EIG121), and mitogen-induced gene-6 (MIG6), three posttranslational EGFR trafficking molecules implicated in EGFR spatiotemporal regulatory pathway. We quantified *MDGI*, *EIG121*, and *MIG6* at mRNA levels by using real-time quantitative RT-PCR in a series of 440 IBCs and at protein levels by using immunohistochemistry in a series of 88 IBCs. Results obtained by RT-PCR showed that in IBCs, *MDGI*, *MIG6*, and *EIG121* mRNA were mainly underexpressed (25.7%, 45.0%, and 16.1%, respectively) particularly in the TNC subtype for *EIG121* (60.3%). We also observed mRNA overexpression of *MDGI* and *EIG121*, respectively, in 12.7% and 22.3% of IBCs. These altered mRNA expressions were confirmed at the protein level. Some links were found between expression patterns of these three genes and several classical pathological and clinical parameters. Only *EIG121* was found to have a prognostic significance (*p* = 0.0038). Altered expression of these three major EGFR posttranslational negative regulators could create an aberrant EGFR-mediated oncogenic signalling pathway in IBCs. MDGI, MIG6, and EIG121 expression status also may be potential useful biomarkers (sensitivity or resistance) in targeted EGFR therapy.

## 1. Introduction

Epidermal growth factor receptor (EGFR) is the founding member of the ErbB receptor tyrosine kinase (RTK) family. RTKs, which contain an extracellular ligand binding domain, a transmembrane domain, and an intracellular tyrosine kinase domain, mediate cellular signal transduction by extracellular ligand binding. The EGFR family of RTKs consists of four members: EGFR/ErbB-1/HER-1, ErbB-2/HER-2/neu, ErbB-3/HER-3, and ErbB-4/HER-4 [1–3]. Upon ligand binding, EGFR family proteins dimerize via receptor homodimerization or heterodimerization and subsequently induce tyrosine kinase activity. Activated EGFR family receptors trigger numerous downstream signalling pathways, such as phosphatidylinositol-3 kinase (PI3K), mitogen-activated protein kinase (MAPK), signal transducer and activator of transcription (STAT), and phospholipase C (PLC), and modulation of calcium channels. These downstream signalling activities control proliferation, mobility and differentiation in morphogenesis, homeostasis, and wound healing. The crucial role of EGFR in these physiological events is evidenced by the embryonic lethality of EGFR knockout animals and tissue defects in EGFR ligand knockout animals [4, 5]. Many carcinomas, including those affecting the lung, colon, and kidney, are characterized by overexpression and/or gene alteration that activate EGFR [6–8]. EGFR signalling activation has been linked with resistance to cytotoxic drugs, hormone, and anti-EGFR therapies and is an indicator of poor prognosis. In IBCs and particularly the TNC subtype, clinical trials using anti-EGFR therapies are actually performed although no activating alterations, including EGFR mutations, amplifications, or rearrangements have been clearly identified. Moreover, EGFR expression levels in IBCs have not been shown to correlate with cancer responsiveness and recent data have suggested that *EGFR* mRNA is not frequently overexpressed as previously reported but surprisingly mainly underexpressed compared with normal tissues [9]. Therefore, a complete understanding of EGFR functions has important implications in cancer biology. Furthermore, the identification of regulatory mechanisms and molecular basis of sensitivity/resistance to EGFR inhibitors will help to establish a rational basis for targeted therapies.

EGFR signalling is a highly regulated process with a tight balance between activation and inactivation of the receptor. However, this process is much more complex than it first seemed due to various mechanisms recently identified that regulate EGFR signalling. Numerous molecular mechanisms classically impact EGFR signalling, including ligand concentration, receptor density, duration of activated receptors, and proximity of EGFR to downstream effectors. Among them, the endocytic pathway has recently emerged as a key spatiotemporal regulator of EGFR [10]. To reduce the level of a specific plasma membrane protein in a short period, cells internalize the protein from the cell surface by endocytosis and degrade it in the lysosomal compartment. It is now widely accepted that “endocytic matrix” is a master organizer of signalling, governing resolution of signals in space and time. Consequently, endocytosis affects crucial cell functions that range from proliferation to cell motility. Recent data suggest that cancer could be related to alteration of subcellular protein localization, trafficking, and compartmentalization [11]. At least three mechanisms have been proposed to explain how endocytic trafficking pathway deregulation could contribute to malignant transformation, including receptor dephosphorylation, removing receptor from cell surface, and targeting ligand-receptor complex for degradation. To explore the endocytic pathway in IBCs, we analyzed three major EGFR trafficking molecules implicated in EGFR sequestration (mammary-derived growth inhibitor (MDGI) also called FABP3), EGFR endolysosomal degradation (estrogen-induced gene-121 (EIG121) also called KIAA1324), and EGFR inhibition/dephosphorylation (mitogen-induced gene-6 (MIG6) also called ERRFI1).

The aim of this study was to identify new targeted modalities for IBC treatments by quantifying *MDGI*, *EIG121*, and *MIG6* mRNA levels in a series of 440 IBCs from patients with known clinical and pathological status and long-term outcome. We also analyzed MDGI, EIG121, and MIG6 at protein levels in a series of 88 IBCs.

## 2. Materials and Methods

### 2.1. Patients and Samples

We obtained tumor samples from 440 patients treated at Curie Institute, Rene Huguenin Hospital, (Saint-Cloud, France) from 1978 to 2008. All patients who entered our institution before 2007 were informed that their tumor samples might be used for scientific purposes and had the opportunity to decline. Since 2007, patients entering our institution have given their approval also by signed informed consent. Tumor samples containing more than 70% of tumor cells were considered suitable for analysis. Immediately after surgery, the tumor samples were placed in liquid nitrogen until RNA extraction. The patients met the following criteria: primary unilateral nonmetastatic breast carcinoma; complete clinical, histological, and biological information available; no preoperative radiotherapy or chemotherapy; and complete follow-up at our institute. Patients underwent physical examination every 3 months for 2 years, then annually. Mammograms were performed annually. Median follow-up was 8.9 years (range 6 months to 29 years).

Ten specimens of adjacent normal breast tissue from breast cancer patients on normal breast tissue from women undergoing cosmetic breast surgery were used as sources of normal RNA.

The histological type and the number of positive axillary nodes were established at the time of surgery. IBCs were scored according to Scarff, Bloom, and Richardson (SBR) histopronostic system. Hormone receptor (HR) estrogen and progesterone receptors (ER and PR) were routinely analyzed at the time of diagnosis on frozen tumors using ligand binding assay until 1988, enzyme immunoassay (ER-EIA Monoclonal, PgR-EIA Monoclonal, Abbott Laboratories, Abbott Park, IL) between 1988 and 2000, and then immunohistochemistry on paraffin sections. Tyrosine kinase receptor HER2 status was routinely analyzed by immunohistochemistry (with confirmation by FISH of the 2+ cases). In this study, the tumor subtype status was confirmed by RT-PCR on frozen samples [12, 13]. According to HR (ER and PR) and HER2 status, the total population (*n* = 440) was subdivided in 4 subtypes: subtype 1 (HR+ (ER+ or/and PR+) and HER2+) (*n* = 50); subtype 2 (HR+ (ER+ and/or PR+) and HER-) (*n* = 281); subtype 3 (HR- (ER- and PR-) and HER2+) (*n* = 46); and subtype 4 that corresponds to the TNC subtype (ER-, PR- and HER2-) (*n* = 63).

### 2.2. RNA Extraction

Total RNA was extracted from breast tissue samples by using acid-phenol guanidinium as previously described [14]. RNA quality was determined by electrophoresis through agarose gels, staining with ethidium bromide, and visualization of the 18S and 28S RNA bands under ultraviolet light.

### 2.3. Real-Time RT-PCR

Quantitative values were obtained from the cycle number (Ct value) at which the increase in the fluorescence signal associated with exponential growth of PCR products started to be detected by the laser detector of the ABI Prism 7900 sequence detection system (Perkin-Elmer Applied Biosystems, Foster City, CA), using PE Biosystems analysis software according to the manufacturer's manuals. The precise amount of total RNA added to each reaction mix (based on optical density) and its quality (i.e., lack of extensive degradation) are both difficult to assess. Therefore, transcripts of the *TBP* gene (GenBank accession NM_003194) encoding the TATA box-binding protein (a component of the DNA-binding protein complex TFIID) were also quantified as an endogenous RNA control. Each sample was normalized on the basis of its *TBP* content. *TBP* was selected as an endogenous control due to the moderate prevalence of its transcripts and the absence of known *TBP* retropseudogenes (retropseudogenes lead to coamplification of contaminating genomic DNA and thus interfere with RT-PCR, despite the use of primers in separate exons) [13]. Results, expressed as *N*-fold differences in target gene expression relative to the *TBP* gene and termed “N*target*,” were determined as N*target* = 2^ΔCtsample^, where the ΔCt value of the sample was determined by subtracting the average Ct value of the target gene from the average Ct value of the *TBP* gene. In these mRNA and protein series, we have previously analyzed EGFR expression [9]. *MDGI*, *EIG121*, and *MIG6* mRNA expressions were normalized to the housekeeping gene *TBP* (which encodes the TATA box-binding protein), and the median relative expression level in the 10 normal breast tissues was attributed a value of 1. *MDGI*, *EIG121*, and *MIG6* mRNA expression levels in the 10 normal breast samples were 0.6 to 1.6. The threshold value of 2 was then considered overexpression and ≤0.5 underexpression in breast cancer tissues, as reported in previous studies [9, 15, 16]. The conditions of cDNA synthesis and PCR were as previously described [13].

### 2.4. Immunohistochemistry

Formalin-fixed and paraffin-embedded tissue blocks, obtained at the time of initial diagnosis, were retrieved from of the Department of Pathology archives. Sections of 3 *μ*m in thickness were cut with a microtome from the paraffin-embedded tissue blocks of normal breast tissue and IBCs. Tissue sections were deparaffinised and rehydrated through a series of xylene and ethanol washes. Immunohistochemical assays were performed in the series of 88 IBCs: subtype 1 (HR+ (ER+ or/and PR+) and HER2+) (*n* = 13); subtype 2 (HR+ (ER+ and/or PR+) and HER-) (*n* = 47); subtype 3 (HR- (ER- and PR-) and HER2+) (*n* = 10); and subtype 4 (ER-, PR- and HER2-) (*n* = 18) using a panel of antibodies against EIG121 (polyclonal anti-EIG121, dilution 1/300, Novus Biologicals™); MDGI (polyclonal anti-MDGI, dilution 1/50, Spring Bioscience™); and MIG6 (polyclonal anti-ERRFI1, dilution 1/50, Proteintech™). Positive IHC reactions were defined as a cytoplasmic staining for EIG121, MDGI, and MIG6.

According to manufactory datasheet, positive immunohistochemistry control was performed using normal endometrial tissue, colon adenocarcinoma, and tonsillitis tissue for EIG121, MDGI, and MIG6, respectively. In breast tissue, we used terminal ductulolobular units (TDLU) as internal positive control, and we verified that antigen retrieval was utilized correctly, all reagents were mixed and applied properly, antibody dilutions and the method of staining were correct, and the incubation times and temperatures were optimal. We also performed both negative reagent and internal negative controls to ensure specificity and sensitivity of the three antibodies and rule out additional or nonspecific staining when the primary antibody is removed from the protocol.

In our series, a protein IHC intensity score (IHC score 0 to 3) was attributed by comparing neoplastic cells to normal epithelial cells of TDLU.

### 2.5. Statistical Analysis

The distributions of target (mRNA and protein) expression levels were characterized by their median values and ranges. Relationships between mRNA levels of the different target genes, and between mRNA levels and clinical parameters, were identified by using nonparametric tests, namely, the chi-square test (relation between 2 qualitative parameters), and the Spearman rank correlation test (relation between 2 quantitative parameters). Differences were considered significant at confidence levels greater than 95% (*p* < 0.05).

Metastasis-free survival (MFS) was determined as the interval between initial diagnosis and detection of the first metastasis. Survival distributions were estimated by the Kaplan-Meier method, and the significance of differences between survival rates was ascertained with the log-rank test.

## 3. Results

**Figure 1:**
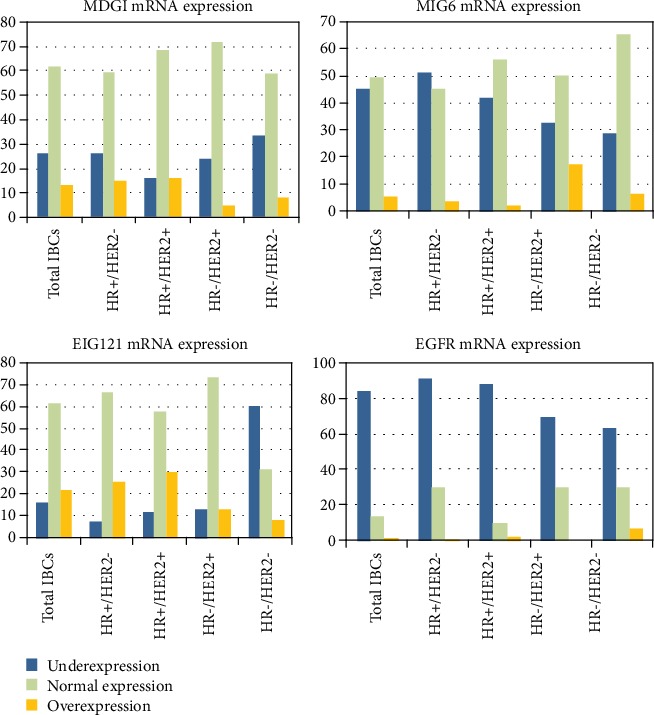
Results obtained by RT-PCR showed that *MIG6* is mainly underexpressed in invasive breast carcinomas and more particularly in the HR+/HER2- subtype. Both overexpression and underexpression were observed for the two other genes: *MDGI* and *EIG121* are mainly overexpressed in the two HR+ subtypes. TNCs showed high frequency of *EIG121* underexpression.

Results obtained by RT-PCR (Figure 1) showed that *MIG6* was mainly underexpressed in our breast tumor series (underexpression 45.0%, overexpression 5.5%; median mRNA level, 0.53; range, 0.00-5.93) and more particularly in the HR+/HER2- subtype (51.2%). Both overexpression and underexpression were observed for the two other genes: *MDGI* (underexpression 25.7%, overexpression 12.7%; median mRNA level, 0.76; range, 0.14-26.84), and *EIG121* (underexpression 16.1%, overexpression 22.3%; median mRNA level, 1.19; range, 0.00-15.73). *MDGI* and *EIG121* were mainly overexpressed in the two HR+ subtypes. TNCs showed high frequency of *EIG121* underexpression (60.3%).  Table 1 also shows mRNA levels of *EGFR* previously analyzed in the same series of 440 IBCs [9]. *EGFR* mRNA was underexpressed in IBCs relative to normal breast tissues (HR+/HER2+: 88.0%; HR+/HER2-: 91.5%; HR-/HER2+: 69.6%; and HR-/HER2-: 63.5%). *EGFR* mRNA was overexpressed in only 6.3% of triple-negative (HR-/HER2-) tumors and in almost none of the tumors in the other 3 subtypes (Table 1).

**Figure 2:**
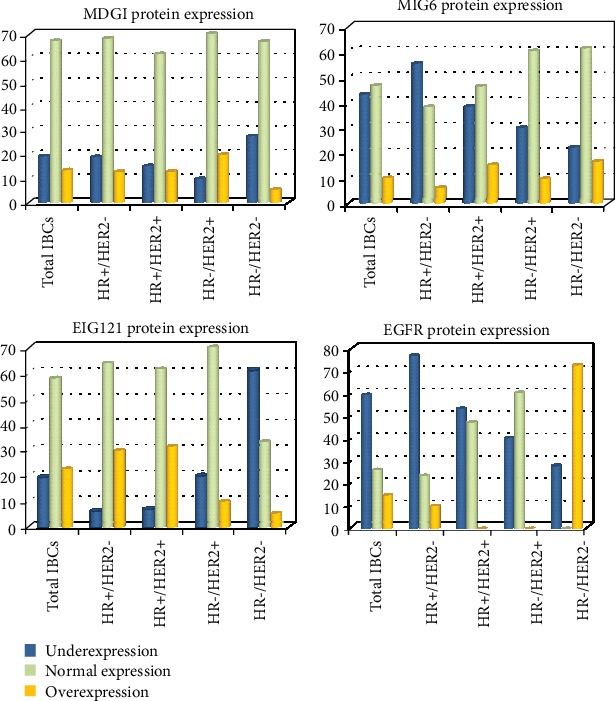
Results of MDGI, MIG6, and EIG121 confirmed at a protein level by using IHC in a population of invasive breast carcinomas (*n* = 88). Protein underexpression of the 3 inhibitors is identified in 19.4% (MDGI), 43.2% (MIG6) and 19.4% (EIG121). Interestingly, EIG121 underexpression is observed in 61.1% of the TNC subtype and MIG6 underexpression in 55.3% of the HR+/HER2- subtype.

**Figure 3:**
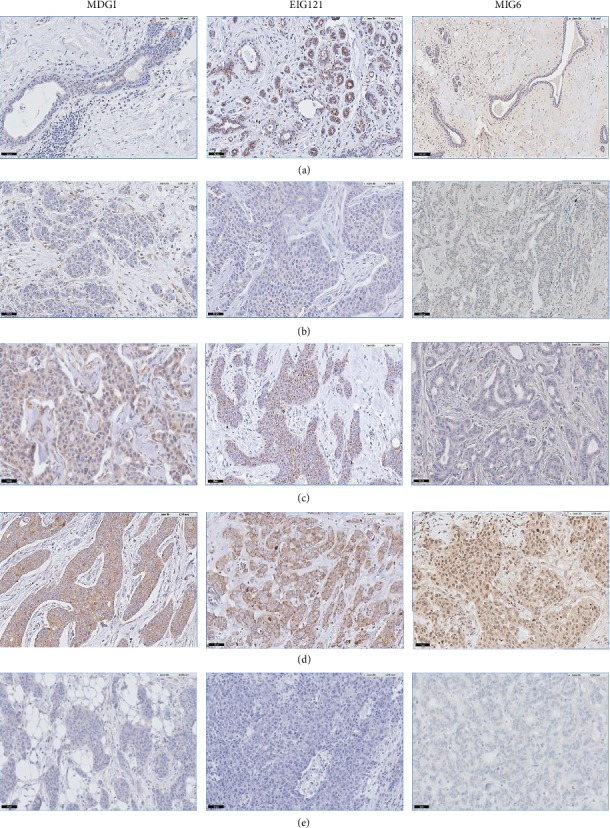
MDGI, EIG121, and MIG6 immunohistochemical score (IHC) in terminal ductulolobular units (TDLU) and invasive breast carcinomas (IBC). (a) Normal TDLU; (b) IBC: IHC score 1; (c) IBC: IHC score 2; (d) IBC: IHC score 3; (e) IBC: IHC score 0.

MDGI, MIG6, and EIG121 altered expressions were confirmed at the protein level by using IHC in the total population of IBCs tested (*n* = 88) and in the different subtypes. In our series of 88 IBCs, protein underexpression (IHC score 0) of the 3 inhibitors was identified in 19.4% (MDGI), 43.2% (MIG6), and 19.4% (EIG121). Interestingly, we could confirm that EIG121 underexpression was observed in 61.1% of IBCs of the TNC subtype and MIG6 underexpression in 55.3% of HR+/HER2- IBCs (Table 2 and Figures 2 and 3). These similar results obtained both at the mRNA and protein levels suggest that regulation of these 3 genes is mainly transcriptional.

To test the relationship between *EGFR*, *MDGI*, *MIG6*, and *EIG121* gene mRNA levels in the series of 440 IBCs, we used the Spearman rank correlation test for continuous variables (Supplementary Table 1S) and only found a weak positive correlation (*r* = +0.094, *p* = 0.045) between *EGFR* and *MDGI* mRNA levels, reflecting very independent interplay across this four-gene set.

We sought links between *MDGI*, *MIG6*, and *EIG121* qualitative mRNA status and standard clinical, pathological, and biological factors. In IBCs, determination of PIK3CA status is important for diagnosis, prognosis, prediction of resistance, and theranostics. *PIK3CA* mutations are particularly frequent in HR+ breast carcinomas and potent drivers of carcinogenesis through AKT activation, evasion of apoptosis, and promotion of invasion. *PIK3CA* somatic mutations are correlated with significantly better clinical outcome in early-stage IBCs and are associated with resistance to paclitaxel, trastuzumab, and endocrine treatment [17]. We observed in our series significant positive correlation between EIG121 transcripts and *PIK3CA* mutation status (*p* = 0.00067) (Supplementary Tables 2S–4S, respectively). *MDGI* mRNA level was highly associated with lymph node status (Supplementary Table 2S). *MIG6* mRNA level was markedly associated with lymph node status, ER status, and molecular subtypes (Supplementary Table 3S). *EIG121* mRNA level was markedly associated with SBR histological grade, lymph node status, ER and PR status, molecular subtypes, and *PIK3CA* mutation status (Supplementary Table 4S). One simple explanation to the observed positive association between high mRNA level of *EIG121* and *PIK3CA* mutation is that high mRNA level of *EIG121* is also associated with estrogen receptor-positive tumors which are known to have a high proportion of *PIK3CA* mutations [16].

**Figure 4:**
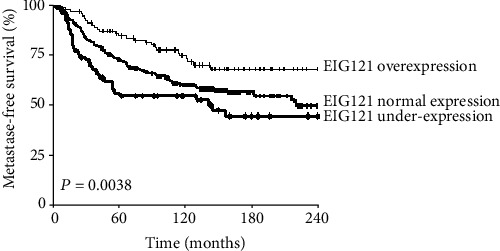
Relations between metastasis-free survival (MFS) and *MDGI*, *MIG6*, and *EIG121* gene mRNA levels. The difference in MFS among patients with downregulated, normal, and upregulated *EIG121* expression was statistically significant (*p* = 0.0038).

Finally, we used a log-rank test to identify relations between metastasis-free survival (MFS) and *MDGI*, *MIG6*, and *EIG121* gene mRNA levels. The difference in MFS among patients with downregulated, normal, and upregulated *EIG121* expression was statistically significant (*p* = 0.0038) (Figure 4). Patients with the poorest prognosis showed *EIG121* underexpression (5-year RFS 56.9% (50.9-62.9); 15-year RFS 45.3% (38.6-52.1)), while those with the best prognosis had *EIG121* overexpression (5-year RFS 85.4% (81.8-89.0); 15-year MFS 68.8% (63.5-74.9)). The third subgroup with normal *EIG121* expression had an intermediate outcome (5-year MFS 73.4% (70.7-76.1); 15-year MFS 57.1% (53.8-60.5)). MFS was not influenced by expression status of the two other genes, *MDGI* and *MIG6*.

## 4. Discussion

**Figure 5:**
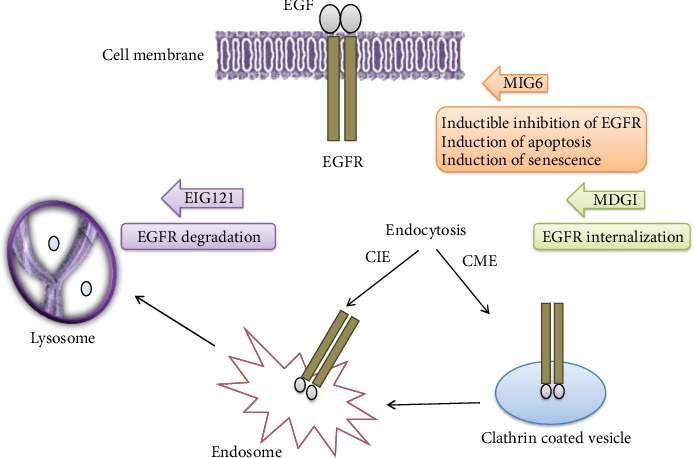
EGFR posttranslational regulators MDGI, MIG6, and EIG121 signalling pathways. EGF binding causes ligand-receptor complex to internalize *via* clathrin. Liganded EGFR monomers dimerize and translocate along the plasma membrane until association with a membrane domain that is enriched with clathrin on the intracellular face. This domain invaginates to initiate a clathrin-coated pit, which pinches off to form a clathrin-coated vesicle. Clathrin is shed from this primary vesicle to produce an intermediate vesicle that fuses with and delivers EGF/EGFR complex to an early endosome. The early endosome then matures into a late endosome and delivers cargo to lysosome.

For over 25 years, the field has evolved from viewing endocytosis as strictly a negative regulator of the ligand receptor/complex to now appreciating its complexity in both positively and negatively modulating receptor-effector communications [11]. In addition to initiating activation of downstream signalling pathways, EGF binding also causes ligand-receptor complex to internalize via clathrin or less frequently caveolae or various small molecular weight GTP-binding proteins (Arf6, RhoA, and Cdc42). Liganded EGFR monomers dimerize and translocate along the plasma membrane until association with a membrane domain that is enriched with clathrin on the intracellular face. This domain invaginates to initiate a clathrin-coated pit, which pinches off to form a clathrin-coated vesicle. Clathrin is shed from this primary vesicle to produce an intermediate vesicle that fuses with and delivers EGF/EGFR complex to an early endosome (Figure 5). Ligand/receptor complex is then readied for its ultimate cellular fate: (i) the early endosome matures into a late endosome and delivers cargo to a lysosome, (ii) recycling endosome pinches off the early endosome and ligand and receptor recycle back to plasma membrane, or (iii) an endosome forms to deliver receptor to some other intracellular organelles (mitochondria and nucleus via the trans-Golgi network and endoplasmic reticulum) [18].

Each endocytic route has a very different consequence on EGFR signalling. Traffic to lysosomes results in attenuated signalling, due to receptor degradation. Recycling back to the plasma membrane allows EGFR restimulation by extracellular ligands. Transporting via the Golgi apparatus and endoplasmic reticulum to the nucleus allows EGFR to act as a transcriptional regulator involved in cell proliferation, tumor progression, DNA repair, and chemo- and radioresistance [19]. Moreover, recent data have demonstrated that the endocytic pathway is critical for appropriate spatial localization of ligand-receptor complex to activate downstream effectors. Inhibition of EGFR endocytosis decreases efficiency of signalling to MAPK and PI3K and induction of apoptosis. Further, maintaining active EGFR at the plasma membrane enhances phosphorylation of EGFR and DNA synthesis. Thus, this trafficking endomembrane system determines the strength and duration of signalling responses not only by controlling sorting events such as recycling and transporting to a lysosomal compartment for ligand/receptor degradation but also by recruiting downstream effectors of signalling complexes.

We previously showed that EGFR was markedly underexpressed both at the mRNA and protein levels in IBCs relative to normal breast tissues with some overexpression in the TNC subtype [9], confirming some data [20]. In the present study, in order to further explore EGFR spatiotemporal regulation by the endocytic network, we selected and analyzed 3 molecules implicated in EGFR sequestration, degradation, and inhibition (i.e., *MDGI*, *MIG6*, and *EIG121*) in a series of 440 unilateral IBCs from patients with known clinical, pathological, and biological (including *PIK3CA* mutation and *EGFR* expression) status and long-term outcome.

MDGI (also known as FABP3) is a small 15 kDa protein that belongs to the family of fatty acid-binding proteins (FABPs). This cytosolic molecule plays a role in differentiation of epithelial cells [21, 22]. The *MDGI* gene is silenced by hypermethylation in human breast cancer cell lines and in some primary breast carcinomas, suggesting a tumor suppressor role [23]. In breast cancer cell lines, MDGI plays an important role in EGFR subcellular relocalization into an intracellular pool where the receptor is active but in a compartment that renders anti-EGFR antibody therapy inefficient [3]. MDGI interacts with integrin alpha subunits and suppresses integrin activity and cell migration and invasion [24]. In our series, we documented *MDGI* underexpression in IBCs (25.7% by RT-PCR and 19.4%, by IHC) and more particularly in the TNC subtype (33.3% by RT-PCR and 27.7%, by IHC). Nevertheless, we also observed mRNA *MDGI* overexpression in IBCs (12.7% at the mRNA level and 13.6% at the protein level). *MDGI* overexpression that induces EGFR intracellular accumulation may represent a potentially new molecular mechanism of resistance when using EGFR antibodies.

MIG6 (also known as RALT) is a cytosolic protein whose centrally located ErbB-binding region (EBR) allows specific binding to members of the ErbB receptor family, including ERBB2/HER2 [25]. *MIG6* gene maps on human chromosome 1p36, one of the regions most often targeted by genomic alterations in human tumors [26]. MIG6 suppresses EGFR receptor signalling through (i) catalytic inhibition in an inactive allosteric configuration through its EBR, (ii) receptor downregulation through RALT endocytic domain (RED) interactions with endocytic proteins AP2 and intersectins, (iii) clathrin-mediated endocytosis (CME) to late endosomes and sorting through its binding to the SNARE protein syntaxin 8 (STX8), and (iv) downstream inhibition of EGFR signalling, including activation of ERKs and AKT as well as proliferation and cell mobility [27–37]. MIG6 has an important suppressive function since knockout mice are highly susceptible to cancer formation and MIG6 expression is lost in various cancer cells [27]. Moreover, MIG6 is one of the EGFR inducible feedback inhibitors (IFIs). IFIs have been recently identified as important regulators that reduce EGFR activity and expression at the cell surface. IFIs are actually considered tumor suppressors and gatekeepers of EGFR mitogenic signalling and antagonists of EGFR-driven tumorigenesis [34].

In mammary glands, ductal morphogenesis involves both epithelial cell autonomous, microenvironmental, and paracrine factors. Recent research on mammary gland's development in the *ERRFI1* null mice showed that treatment of mammary epithelial cells with specific ErbB inhibitors AG825 (ErbB2), gefitinib (EGFR), or lapatinib (EGFR and ErbB2) prior to EGF deprivation failed to rescue the EGF-independent survival of *ERRFI1* (encoding *MIG6*) cells [30]. MIG6 has also a p73-dependant proapoptotic role in breast morphogenesis [31]. Thus, *MIG6* regulates mammary epithelial cell death independent of its role as a negative regulator of the ErbB receptors. MIG6 has also been implicated in replicative senescence, and MIG6 or pharmacological-driven EGFR inhibition promotes senescence of HPV-16-infected cervical epithelial cells [38]. These results suggest that MIG6 could variably influence cellular homeostasis through growth inhibition, apoptosis, and senescence induction [38–41]. A recent study confirms that lung cancer stem cells inversely express *EGFR* and *MIG6* genes [42]. In endometrial carcinoma, MIG6 plays a tumor-suppressor role by promoting epithelial cell apoptosis through the inhibition of ERK2 phosphorylation [33]. Moreover, MIG6 orchestrate epithelial-mesenchymal transition-associated kinase switch and mediate the reduction of EGFR [35].

In the present study, we found that *MIG6* expression level was underexpressed with a high frequency in IBCs (45% by RT-PCR and 43.2% by IHC). *MIG6* underexpression was associated with lymph node and ER positivity. To our knowledge, this is the first description of frequencies and patterns of expression of *MIG6* in a large panel of IBCs. MIG6/EGFR ratio could be tested as a new biomarker for predicting TKI response in breast carcinomas.

EIG121 was initially found overexpressed in estrogen-dependent endometrial cancer cell lines, leading to inhibition of cell growth and apoptosis. More recent data have identified EIG121 as a transmembrane protein associated with plasma membrane and trans-Golgi/late endosomal-lysosomal compartments enhancing lysosomal degradation of long-lived proteins [43]. Moreover, EIG121 may have important role in autophagy after nutrient deprivation and exposure to cytotoxic chemotherapeutic agents by linking EGFR with the autophagosome marker microtubule-associated protein light chain-3 (LC3). During autophagy, EIG121 and LC3 translocate into the same autophagosome vesicles and are degraded by a lysosomal mechanism. A previous mechanistic study revealed that EIG121 is located to the late endosome-lysosome compartments and regulates autophagy, a cellular prosurvival mechanism activated when a cell is stressed by lack of nutrients or chemotherapy. It was shown that EIG121 conferred protection against induction of apoptosis on cells exposed to serum starvation and treatment with taxane chemotherapy [44]. In IBCs, *EIG121* overexpression predicts improved survival, in agreement with the present study (Figure 1). Our results also showed high frequency of *EIG121* underexpression in the TNC subtype (60.3% by RT-PCR and 61.1% by IHC), which has generally a poor prognosis. *EIG121* underexpression was also associated with SBR histological grade III and ER and PR negativity.

Taken together, our current results on EGFR and endosomal pathway in IBCs have revealed frequent altered expressions of the three endocytic pathway regulators MDGI, EIG121, and MIG6, respectively, implicated in EGFR internalization, degradation, and inducible inhibition. These results underline the growing importance of posttranslational trafficking molecules in EGFR regulation and highlight the emerging role of EGFR IFIs as antagonists of EGFR-driven tumorigenesis. Altered expression of these three EGFR posttranslational regulators could be sufficient to deregulate inhibition and endolysosomal degradation leading to deficient termination of EGFR signalling. Moreover, implication of EIG121 in autophagy and MIG6 in apoptosis and senescence could potentially create an aberrant EGFR-mediated oncogenic signalling pathway in IBCs, irrespective of EGFR levels of expression.

In conclusion, these data demonstrate a complex pattern of EGFR posttranslational regulator expression and suggest that MDGI, MIG6, and EIG121 expression statute may then represent useful biomarkers, particularly in triple-negative invasive breast carcinomas. Among these three molecules, *EIG121* expression is significantly correlated with MFS and could thus represent a pertinent biomarker for EGFR-targeted therapy.

## Figures and Tables

**Table 1:** *MDGI*, *MIG6*, *EIG121*, and *EGFR* mRNA expression by RT-PCR in breast tumors (*n* = 440).

Genes	mRNA	All tumors *n* = 440 <0.5 *N* >2	HR-/HER2- (TNC) *n* = 63 <0.5 *N* >2	HR-/HER2+ *n* = 46 <0.5 *N* >2	HR+/HER2- *n* = 281 <0.5 *N* >2	HR+/HER2+ *n* = 50 <0.5 *N* > 2	*p* value^∗^
*MDGI*	Status	25.7% 61.6% 12.7%	33.3% 58.8% 7.9%	23.9% 71.8% 4.3%	26.0% 59.4% 14.6%	16.0% 68.0% 16.0%	0.14 (NS)
	Level	0.76 (0.14-26.84)	0.62 (0.15-26.84)	0.83 (0.20-4.78)	0.75 (0.14-18.16)	0.96 (0.29-7.73)	

*MIG6*	Status	45.0% 49.5% 5.5%	28.6% 65.1% 6.3%	32.6% 50.0% 17.4%	51.2% 44.9% 3.9%	42.0% 56% 2.0%	0.0001
	Level	0.53 (0.00-5.93)	0.71 (0.08-3.96)	0.66 (0.13-5.93)	0.49 (0.00-5.34)	0.54 (0.00-3.86)	

*EIG121*	Status	16.1% 61.6% 22.3%	60.3% 31.8% 7.9%	13.0% 74.0% 13.0%	7.5% 66.9% 25.6%	12.0% 58.0% 30.0%	<0.0001
	Level	1.19 (0.00-15.73)	0.33 (0.00-4.53)	0.93 (0.21-4.41)	1.36 (0.08-15.73)	1.49 (0.23-6.65)	

*EGFR*	Status	84.8% 13.8% 1.4%	63.5% 30.2% 6.3%	69.6% 30.4% 0.0%	91.5% 8.1% 0.4%	88% 10.0% 2.0%	
	Level	0.16 (0.01-82.84)	0.44 (0.03-82.84)	0.20 (0.04-1.67)	0.13 (0.01-7.30)	0.15 (0.03-2.58)	

^∗^Chi-squared test: total *p* value (*n* = 440).

**Table 2:** MDGI, MIG6, EIG121, and EGFR protein expression by IHC in breast tumors (*n* = 88).

Protein	All tumors *n* = 88 0 1 2/3	HR-/HER2- (TNC) *n* = 18 0 1 2/3	HR-/HER2+ *n* = 10 0 1 2/3	HR+/HER2- *n* = 47 0 1 2/3	HR+/HER2+ *n* = 13 0 1 2/3	*p* value^∗^
MDGI	19.4% 67% 13.6%	27.7% 66.6% 5.7%	10% 70% 20%	19.1% 68% 12.9%	15.3% 61.7% 23%	0.77 (NS)
MIG6	43.2% 46.6% 10.2%	22.2% 61.1% 16.7%	30% 60% 10%	55.3% 38.2% 6.5%	38.4% 46.3% 15.3%	0.27 (NS)
EIG121	19.4% 57.9% 22.7%	61.1% 33.3% 5.6%	20% 70% 10%	6.3% 63.8% 29.9%	7% 61.5% 31.5%	<0.0001
EGFR	59.1% 26.1% 14.8%	27.7% 0% 72.3%	40% 60% 0%	76.5% 23.5% 0%	53.0% 47.0% 0%	

^∗^Chi squared test: total *p* value (*n* = 88). IHC score 0: underexpression; score 1: normal expression; score 2/3: overexpression.

## Data Availability

The data used to support the findings of this study are available from the corresponding author upon request.

## Supplementary Materials

Supplementary 1 Table 1S: relationship between *EGFR*, *MDGI*, *MIG6*, and *EIG121* gene mRNA levels in the 440 IBCs.

Supplementary 2 Table 2S: relationship between *MDGI* transcript levels and classical clinical biological parameters in a series of 440 breast cancer.

Supplementary 3 Table 3S: relationship between *MIG6* transcript levels and classical clinical biological parameters in a series of 440 breast cancer.

Supplementary 4 Table 4S: relationship between *EIG121* transcript levels and classical clinical biological parameters in a series of 440 breast cancer.
