# Supplementary material for: Altered Expression of Three EGFR Posttranslational Regulators MDGI, MIG6, and EIG121 in Invasive Breast Carcinomas
Source: Anal Cell Pathol (Amst). 2020 Apr 20;2020:9268236. doi: 10.1155/2020/9268236 (PMC7189325; doi:10.1155/2020/9268236)
Supplement: Supplementary 2 — Table 2S: relationship between MDGI transcript levels and classical clinical biological parameters in a series of 440 breast cancer. [file 9268236.f2.zip › Supplementary Table 2S Meseure et al.doc]

**Supplementary Table 2S: Relationship between *MDGI* transcripts levels and classical clinical biological parameters in a series of 440 breast cancer.**

|  |  | Number of patients (%) | | | | |  | |  |
| --- | --- | --- | --- | --- | --- | --- | --- | --- | --- |
|  | Total population (%) | ***MDGI* underexpression** | | ***MDGI***  **normal expression** | | ***MDGI***  ***over*expression** | | *p^a^* | |
|  |  |  |  | |  | |  | |  |
| *Total* | 440 (100.0) | 113 (25.7) | 271 (61.6) | | 56 (12.7) | |  | |  |
|  |  |  |  | |  | |  | |  |
| *Age*  ≤50  >50 | 91 (20.7)  349 (79.3) | 14 (15.4)  99 (28.4) | 61 (67.0)  210 (60.2) | | 16 (17.6)  40 (11.5) | | **0.024** | |  |
| *SBR histological grade*^b,c^  I  II  III | 54 (12.5)  224 (52)  153 (35.5) | 8 (14.8)  65 (29.0)  37 (24.2)  3 | 40 (74.1)  127 (56.7)  99 (64.7)  5 | | 6 (11.1)  32 (14.3)  17 (11.1)  1 | | 0.14 (NS) | |  |
| *Lymph node status*^d^  0  1-3  >3 | 112 (25.5)  230 (52.4)  97 (22.1) | 32 (28.6)  69 (30)  12 (12.4) | 67 (59.8)  127 (55.2)  76 (78.4)  1 | | 13 (11.6)  34 (14.8)  9 (9.3) | | **0.0026** | |  |
| *Macroscopic tumor size*^e^  ≤25mm  >25mm | 212 (49)  221 (51) | 57 (26.9)  54 (24.4)  2 | 128 (60.4)  138 (62.4)  5 | | 27 (12.7)  29 (13.1) | | 0.84 (NS) | |  |
| *ERα status*  Negative  Positive | 114 (25.9)  326 (74.1) | 34 (29.8)  79 (24.2) | 73 (64.0)  198 (60.7) | | 7 (6.1)  49 (15.0) | | **0.039** | |  |
| *PR status*  Negative  Positive | 190 (43.2)  250 (56.8) | 53 (27.9)  60 (24) | 119 (62.6)  152 (60.8) | | 18 (9.5)  38 (15.2) | | 0.17 (NS) | |  |
| *ERBB2 status*  Negative  Positive | 344 (78.2)  96 (21.8) | 94 (27.3)  19 (19.8) | 204 (59.3)  67 (69.8) | | 46 (13.4)  10 (10.4) | | 0.17 (NS) | |  |
| *Molecular subtypes*  RH- ERBB2-  RH- ERBB2+  RH+ ERBB2-  RH+ ERBB2+ | 63 (14.3)  46 (10.4)  281 (63.9)  50 (11.4) | 21 (33.3)  11 (23.9)  73 (26.0)  8 (16) | 37 (58.7)  33 (71.7)  167 (59.4)  34 (68) | | 5 (7.9)  2 (4.3)  41 (14.6)  8 (16) | | 0.14 (NS) | |  |
| *PIK3CA mutation status*  wild type  mutated | 293 (66.6)  147 (33.4) | 78 (26.6)  35 (23.8) | 172 (58.7)  99 (67.3) | | 43 (14.7)  13 (8.8) | | 0.13 (NS) | |  |

^a^χ2Test

^b^Scarff Bloom Richardson classification.

^c^Information available for 431 patients.

^d^Information available for 439 patients.

^e^Information available for 433 patients.
