# Supplementary material for: Altered Expression of Three EGFR Posttranslational Regulators MDGI, MIG6, and EIG121 in Invasive Breast Carcinomas
Source: Anal Cell Pathol (Amst). 2020 Apr 20;2020:9268236. doi: 10.1155/2020/9268236 (PMC7189325; doi:10.1155/2020/9268236)
Supplement: Supplementary 3 — Table 3S: relationship between MIG6 transcript levels and classical clinical biological parameters in a series of 440 breast cancer. [file 9268236.f3.zip › Supplementary Table 3S Meseure et al.doc]

**Supplementary Table 3S: Relationship between *MIG6* transcripts levels and classical clinical biological parameters in a series of 440 breast cancer**

|  |  | Number of patients (%) | |  |
| --- | --- | --- | --- | --- |
|  | Total population (%) | ***MIG6***  **normal expression** | ***MIG6***  ***under*expression** | *p^a^* |
|  |  |  |  |  |
| *Total* | 440 (100.0) | 242 (55.0) | 198 (45.0) |  |
|  |  |  |  |  |
| *Age*  ≤50  >50 | 91 (20.7)  349 (79.3) | 51 (21.1)  191 (78.9) | 40 (20.2)  158 (79.8) | 0.82 (NS) |
| *SBR histological grade*^b,c^  I  II  III | 54 (12.5)  224 (52)  153 (35.5) | 21 (9)  126 (53.8)  87 (37.2) | 33 (16.8)  98 (49.7)  66 (33.5) | 0.051 (NS) |
| *Lymph node status*^d^  0  1-3  >3 | 112 (25.5)  230 (52.4)  97 (22.1) | 78 (32.4)  115 (47.7)  48 (19.9) | 34 (17.2)  115 (58.1)  49 (24.7) | **0.0015** |
| *Macroscopic tumor size*^e^  ≤25mm  >25mm | 212 (49)  221 (51) | 204 (45.9)  205 (46.1) | 99 (50.5)  97 (49.5) | 0.88 (NS) |
| *ERα status*  Negative  Positive | 114 (25.9)  326 (74.1) | 77 (31.8)  165 (68.2) | 37 (18.7)  161 (81.3) | **0.0018** |
| *PR status*  Negative  Positive | 190 (43.2)  250 (56.8) | 111 (45.9)  131 (54.1) | 79 (39.9)  119 (60.1) | 0.21 (NS) |
| *ERBB2 status*  Negative  Positive | 344 (78.2)  96 (21.8) | 182 (75.2)  60 (24.8) | 162 (81.8)  36 (18.2) | 0.095 (NS) |
| *Molecular subtypes*  RH- ERBB2-  RH- ERBB2+  RH+ ERBB2-  RH+ ERBB2+ | 63 (14.3)  46 (10.4)  281 (63.9)  50 (11.4) | 45 (18.6)  31 (12.8)  137 (56.6)  29 (12) | 18 (9.1)  15 (7.6)  144 (72.7)  21 (10.6) | **0.0027** |
| *PIK3CA mutation status*  wild type  mutated | 293 (66.6)  147 (33.4) | 169 (69.8)  73 (30.2) | 124 (62.6)  74 (37.4) | 0.11 (NS) |

^a^χ2Test

^b^Scarff Bloom Richardson classification.

^c^Information available for 431 patients.

^d^Information available for 439 patients.

^e^Information available for 433 patients.
