# Supplementary material for: Altered Expression of Three EGFR Posttranslational Regulators MDGI, MIG6, and EIG121 in Invasive Breast Carcinomas
Source: Anal Cell Pathol (Amst). 2020 Apr 20;2020:9268236. doi: 10.1155/2020/9268236 (PMC7189325; doi:10.1155/2020/9268236)
Supplement: Supplementary 4 — Table 4S: relationship between EIG121 transcript levels and classical clinical biological parameters in a series of 440 breast cancer. [file 9268236.f4.zip › Supplementary Table 4S Meseure et al.doc]

**Supplementary Table 4S: Relationship between *EIG121* transcripts levels and classical clinical biological parameters in a series of 440 breast cancer.**

|  |  | Number of patients (%) | | |  |
| --- | --- | --- | --- | --- | --- |
|  | Total population (%) | ***EIG121* underexpression** | ***EIG121***  **normal expression** | ***EIG121***  ***over*expression** | *p^a^* |
|  |  |  |  |  |  |
| *Total* | 440 (100.0) | 71 (16.1) | 271 (61.6) | 98 (22.3) |  |
|  |  |  |  |  |  |
| *Age*  ≤50  >50 | 91 (20.7)  349 (79.3) | 21 (23.1)  50 (14.3) | 57 (62.6)  214 (61.3) | 13 (14.3)  85 (24.4) | **0.034** |
| *SBR histological grade*^b,c^  I  II  III | 54 (12.5)  224 (52)  153 (35.5) | 3 (5.6)  28 (12.5)  37 (24.2)  3 | 38 (70.4)  134 (59.8)  95 (62.1)  4 | 13 (24.1)  62 (27.7)  21 (13.7)  2 | **0.00042** |
| *Lymph node status*^d^  0  1-3  >3 | 112 (25.5)  230 (52.4)  97 (22.1) | 26 (23.2)  34 (14.8)  10 (10.3)  1 | 60 (53.6)  137 (59.6)  74 (76.3) | 26 (23.2)  59 (25.7)  13 (13.4) | **0.0045** |
| *Macroscopic tumor size*^e^  ≤25mm  >25mm | 212 (49)  221 (51) | 37 (17.5)  33 (14.9)  1 | 126 (59.4)  141 (63.8)  4 | 49 (23.1)  47 (21.3)  2 | 0.64 (NS) |
| *ERα status*  Negative  Positive | 114 (25.9)  326 (74.1) | 44 (38.6)  27 (8.3) | 58 (50.9)  213 (65.3) | 12 (10.5)  86 (26.4) | **<0.0000001** |
| *PR status*  Negative  Positive | 190 (43.2)  250 (56.8) | 57 (30)  14 (5.6) | 106 (55.8)  165 (66) | 27 (14.2)  71 (28.4) | **<0.0000001** |
| *ERBB2 status*  Negative  Positive | 344 (78.2)  96 (21.8) | 59 (17.2)  12 (12.5) | 208 (60.5)  63 (65.6) | 77 (22.4)  21 (21.9) | 0.52 (NS) |
| *Molecular subtypes*  RH- ERBB2-  RH- ERBB2+  RH+ ERBB2-  RH+ ERBB2+ | 63 (14.3)  46 (10.4)  281 (63.9)  50 (11.4) | 38 (60.3)  6 (13.0)  21 (7.5)  6 (12) | 20 (31.7)  34 (73.9)  188 (66.9)  29 (58) | 5 (7.9)  6 (13.0)  72 (25.6)  15 (30) | **<0.0000001** |
| *PIK3CA mutation status*  wild type  mutated | 293 (66.6)  147 (33.4) | 61 (20.8)  10 (6.8) | 174 (59.4)  97 (66) | 58 (19.8)  40 (27.2) | **0.00067** |

^a^χ2Test

^b^Scarff Bloom Richardson classification.

^c^Information available for 431 patients.

^d^Information available for 439 patients.

^e^Information available for 433 patients.
